# Supplementary material for: Rise in mortality involving poisoning by medicaments other than narcotics, including poisoning by psychotropic drugs in different age/racial groups in the US
Source: PLoS One. 2019 Jul 19;14(7):e0219711. doi: 10.1371/journal.pone.0219711 (PMC6641145; doi:10.1371/journal.pone.0219711)
Supplement: S1 Checklist — (DOC) [file pone.0219711.s001.doc]

STROBE Statement—Checklist of items that should be included in reports of ***cohort studies***

|  | Item No | Recommendation |
| --- | --- | --- |
| **Title and abstract** | 1 | (*a*) Cohort study (statistical analysis of mortality data aggregated by year and race/ethnicity, as well as by year/state in the US) |
|  |  | (*b*) Summary: The Abstract provides a concise summary of the study design, results and conclusions |
| Introduction | | |
| Background/rationale | 2 | The Introduction explains the rise in the rates of mortality involving poisoning by various substances, racial discrepancies in the above rise, differences in consumption of various medications by race, and the need for a granular evaluation of the volume/trends for deaths involving poisoning by medicaments other than narcotics/psychodysleptics |
| Objectives | 3 | The last paragraph of the Introduction explains how the available data are used in our study to examine the trends in mortality involving poisoning by medicaments other than narcotics/psychodysleptics |
| Methods | | |
| Study design | 4 | The Methods section explains how we evaluate the trends in various mortality rates using regression, relative changes in those rates for different time periods, and the spatial variability in the rates of mortality involving poisoning by different substances |
| Setting | 5 | It is mentioned throughout the paper that we use aggregate US mortality data stratified by year/race; study of the spatial variability in the rates of mortality involving poisoning by different substances involves the use of mortality data stratified by age/state |
| Participants | 6 | (*a*) This is a population-level study based on publicly available data for all US deaths involving poisoning by different substance in different age/racial groups (and ages/states in the spatial analysis) during different calendar years |
|  |  | (*b*) N/A (though we draw a comparison between mortality rates in different racial groups) |
| Variables | 7 | The outcomes are mortality rates in different age/racial groups (or different age groups/states) during different years. For the trend analysis, the covariate is the calendar year. |
| Data sources/ measurement | 8* | We state in the Methods section that we utilize the publicly available US CDC Wonder data on death/population counts (estimates), available through ref. 29 (<https://wonder.cdc.gov/wonder/help/ucd.html> ). Those data may be freely and publicly downloaded at the URL provided. |
| Bias | 9 | Some of the potential sources of bias are described in the penultimate paragraph of the Discussion |
| Study size | 10 | This is a population-level study, and the entire US mortality data for each given age/racial group/year, as well as age group/state/year are used in the study |
| Quantitative variables | 11 | We use count data on deaths with different causes present on death certificate (as explained in the Methods) for different age groups/racial groups/years, as well as age groups/states/years |
| Statistical methods | 12 | (*a*) We use regression in the analysis of trends as described in the “Trends in mortality rates (regression analysis)” subsection of the Methods. We use correlation analysis as described in the “Spatial variability in the rates of mortality involving poisoning by different substances” subsection of the Methods. |
|  |  | (*b*) No multivariate methods are used in the paper, with separate analyses performed for each age/racial group |
|  |  | (*c*) There are no missing data in the study |
|  |  | (*d*) The study uses existing, publicly available mortality data; thus no follow-up applies to this study design |
|  |  | (*e*) Sensitivity analyses: we compare trends in different mortality data streams to preclude the possibility of confounding due to mischaracterized mortality data (e.g. potential contributing causes not appearing on death certificates) |
| Results | | |
| Participants | 13* | (a) This is a population-level study with all the US deaths involving poisoning by various substances included. We report that the annual number of deaths involving poisoning by medicaments but not narcotics and psychodysleptics grew from 4,332 for the 2000-2001 period to 11,401 for the 2016-2017 period; annual numbers of deaths for different causes of death used in this study are available through ref. 29 (<https://wonder.cdc.gov/wonder/help/ucd.html> ). Those data may be freely and publicly downloaded at the URL provided. |
|  |  | (b) Give reasons for non-participation at each stage: N/A |
|  |  | (c) Consider use of a flow diagram: Our study evaluates relations between different pairs of variables as described in the Methods, with no changes in the status of participants. We believe that the methodology is presented in detail and no flow diagram is required. |
| Descriptive data | 14* | (a) We use US population-level data stratified by age/racial group/year, or age group/state/year |
|  |  | (b) Indicate number of participants with missing data for each variable of interest: N/A |
|  |  | (c) The study use existing US mortality data; thus follow-up doesn’t apply to this study design |
| Outcome data | 15* | Outcome events are the mortality counts. We report that the annual number of deaths involving poisoning by medicaments but not narcotics and psychodysleptics grew from 4,332 for the 2000-2001 period to 11,401 for the 2016-2017 period and estimate trends in the rates of mortality involving poisoning by various substances (with the different categories of death studied in the this paper described in Table 1). We plot the various mortality rates in Figures 1-5 and estimate the trends in the those mortality rates in Tables 2-6 |
| Main results | 16 | (*a*) We study relations between different pairs of variables that involves no adjusting besides the stratification by age/racial group. Confidence intervals for the annual growth in mortality rates are provided in Tables 2-5 |
|  |  | (*b*) Confidence intervals for the annual growth in mortality rates are provided in Tables 2-5 |
|  |  | (*c*) If relevant, consider translating estimates of relative risk into absolute risk for a meaningful time period: we estimate both the relative growth in mortality rates (Table 6), as well as the absolute growth (Tables 2-5) |
| Other analyses | 17 | Results of the analyses about spatial variability in the rates of mortality involving poisoning by different substances are presented in the Supporting Information |
| Discussion | | |
| Key results | 18 | The key results are summarized in the Discussion (particularly the first paragraph, but also the second paragraph, with a concise summary provided in the last paragraph) |
| Limitations | 19 | The penultimate paragraph of the Discussion describes the potential limitations related to our study and suggests several arguments supporting the notion that our main results cannot be explained by potential biases |
| Interpretation | 20 | The penultimate paragraph of the Discussion describes the potential sources of bias. It is mentioned in several places in the paper that further work is needed to evaluate the full scope of health related outcomes, including mortality associated with the use of different medications. |
| Generalisability | 21 | Our study provides a granular analysis of the US data on mortality involving poisoning by different substances and mentions evidence from the literature about the contribution of medication use to mortality for which poisoning does not appear on the death certificate. One should note that a number of other countries, including the UK and a number of other EU countries have not experienced analogous rises in the rates of mortality involving poisoning. |
| Other information | | |
| Funding | 22 | This study involves no funding |

*Give information separately for exposed and unexposed groups.

**Note:** An Explanation and Elaboration article discusses each checklist item and gives methodological background and published examples of transparent reporting. The STROBE checklist is best used in conjunction with this article (freely available on the Web sites of PLoS Medicine at http://www.plosmedicine.org/, Annals of Internal Medicine at http://www.annals.org/, and Epidemiology at http://www.epidem.com/). Information on the STROBE Initiative is available at http://www.strobe-statement.org.
