# Supplementary material for: Rise in mortality involving poisoning by medicaments other than narcotics, including poisoning by psychotropic drugs in different age/racial groups in the US
Source: PLoS One. 2019 Jul 19;14(7):e0219711. doi: 10.1371/journal.pone.0219711 (PMC6641145; doi:10.1371/journal.pone.0219711)
Supplement: S1 File — (DOCX) [file pone.0219711.s002.docx]

Supporting Information for “Rise in mortality involving poisoning by medicaments other than narcotics, including poisoning by psychotropic drugs in different age/racial groups in the US”.

**Section S1**: Spatial variability in the rates of mortality involving poisoning by psychotropic medications other narcotics and psychodysleptics, and rates of mortality involving poisoning by narcotics and psychodysleptics

As suggested in the Results section in the main body of the text, there were no correlations between state-specific rates of mortality involving poisoning by psychotropic drugs but not narcotics/psychodysleptics (category D in Table 1) and state-specific rates of mortality involving poisoning by narcotics/psychodysleptics (category B in Table 1) during the 2013-2017 period; those correlation were -0.06(95% CI (-0.33,0.22)) for persons aged 45-64y, and -0.16(-0.42,0.12) for persons aged 25-44y. Table S1 lists the 20 states with the highest rates of mortality involving poisoning by psychotropic drugs but not narcotics/psychodysleptics, as well as the 20 states with the highest rates of mortality involving poisoning by narcotics/psychodysleptics per 100,000 persons aged 25-44y during the 2013-2017 period; Table S2 presents the corresponding lists for persons aged 45-64y, with all the data extracted from [1]. Lists of states with the highest rates of mortality involving poisoning by psychotropic drugs but not narcotics/psychodysleptics are quite different from lists of states with the highest rates of mortality involving poisoning by narcotics/psychodysleptics, with a notable presence of western states among those with the highest rates of mortality involving poisoning by psychotropic drugs but not narcotics/psychodysleptics (particularly for persons aged 45-64y) vs. a notable presence of eastern (particularly northeastern) states among those with the highest rates of mortality involving poisoning by narcotics/psychodysleptics. Furthermore, rates of mortality involving poisoning by psychotropic drugs but not narcotics/psychodysleptics are higher for persons aged 45-64y compared to persons aged 25-44y, while rates of mortality involving poisoning by narcotics/psychodysleptics are somewhat higher for persons aged 25-44y compared to persons aged 45-64y.

| Psychotropic drugs but not narcotics/psychodysleptics | |  | Narcotics/psychodysleptics | |
| --- | --- | --- | --- | --- |
| State | Rate per 100,000 |  | State | Rate per 100,000 |
| Oklahoma | 9.78 |  | West Virginia | 83.15 |
| Nevada | 7.88 |  | New Hampshire | 62.85 |
| New Mexico | 7.75 |  | Ohio | 57.67 |
| West Virginia | 6.88 |  | Massachusetts | 48.38 |
| Hawaii | 6.42 |  | Rhode Island | 46.68 |
| Arizona | 6.14 |  | Maine | 44.63 |
| Utah | 5.73 |  | Kentucky | 43.7 |
| Arkansas | 5.17 |  | Connecticut | 40 |
| Montana | 5.06 |  | Maryland | 39.26 |
| Iowa | 4.87 |  | Delaware | 36.93 |
| Alaska | 4.76 |  | New Mexico | 35.43 |
| Missouri | 4.65 |  | Vermont | 35.09 |
| Washington | 4.51 |  | Michigan | 30.24 |
| Idaho | 4.5 |  | Tennessee | 30.08 |
| South Dakota | 4.29 |  | Pennsylvania | 29.89 |
| Kansas | 3.9 |  | Utah | 28.31 |
| California | 3.79 |  | North Carolina | 27.77 |
| Wyoming | 3.68 |  | Missouri | 27.67 |
| Kentucky | 3.63 |  | Wisconsin | 27.07 |
| North Dakota | 3.59 |  | New Jersey | 25.35 |

S1 Table: 20 states with the highest rates of mortality involving poisoning by psychotropic drugs but not narcotics/psychodysleptics, as well as the 20 states with the highest rates of mortality involving poisoning by narcotics/psychodysleptics per 100,000 persons aged 25-44y during the 2013-2017 period (including the corresponding mortality rates).

| Psychotropic drugs but not narcotics/psychodysleptics | |  | Narcotics/psychodysleptics | |
| --- | --- | --- | --- | --- |
| State | Rate per 100,000 |  | State | Rate per 100,000 |
| Hawaii | 16.5 |  | District of Columbia | 77.37 |
| Nevada | 14.04 |  | West Virginia | 55.3 |
| Oklahoma | 13.68 |  | Rhode Island | 39.38 |
| Arizona | 10.56 |  | Ohio | 37.52 |
| Utah | 9.25 |  | Maryland | 36.97 |
| New Mexico | 9.07 |  | Kentucky | 31.28 |
| California | 9.02 |  | Nevada | 30.88 |
| Washington | 7.49 |  | New Mexico | 30.57 |
| Idaho | 6.05 |  | Massachusetts | 30.57 |
| Colorado | 5.96 |  | Connecticut | 30.17 |
| Wyoming | 5.91 |  | Tennessee | 29.8 |
| Alaska | 5.91 |  | Utah | 29.45 |
| Iowa | 5.76 |  | New Hampshire | 28.24 |
| Oregon | 5.65 |  | Delaware | 26.58 |
| West Virginia | 5.27 |  | Oklahoma | 24.65 |
| Kansas | 4.86 |  | Michigan | 22.47 |
| Rhode Island | 4.76 |  | Illinois | 22.3 |
| Missouri | 4.54 |  | Maine | 22.18 |
| Arkansas | 4.25 |  | South Carolina | 21.79 |
| Montana | 4.15 |  | New York | 21.12 |

S2 Table: 20 states with the highest rates of mortality involving poisoning by psychotropic drugs but not narcotics/psychodysleptics, as well as the 20 states with the highest rates of mortality involving poisoning by narcotics/psychodysleptics per 100,000 persons aged 45-64y during the 2013-2017 period (including the corresponding mortality rates).

**Reference**

[1] US CDC Wonder. Underlying cause of death 1999-2017. 2018. Updated on Dec. 6, 2018. Accessed on Feb. 1, 2019. Available from: <https://wonder.cdc.gov/wonder/help/ucd.html>
